# Non-coding RNAs: Emerging contributors to chemoresistance in chronic myeloid leukemia

**DOI:** 10.1016/j.lrr.2025.100513

**Published:** 2025-05-09

**Authors:** Laya Ghadyani nejhad, Mahsa Sohani, Nasrin Alizad Ghandforoush, Mohsen Nikbakht, Saeed Mohammadi, Mohammad Vaezi, Shahrbano Rostami, Bahram Chahardouli

**Affiliations:** a Hematologic Malignancies Research Center, Research Institute for Oncology, Hematology and Cell Therapy, Tehran University of Medical Sciences, Iran; b Student Research Committee, Department of Hematology and Blood Banking, School of Allied Medical Sciences, Shahid Beheshti University of Medical Sciences, Tehran, Iran; c Cell Therapy and Hematopoietic Stem Cell Transplantation Research Center, Research Institute for Oncology, Hematology and Cell Therapy, Tehran University of Medical Sciences, Iran; d Department of Internal Medicine, School of Medicine, Hematology, Oncology and Stem Cell Transplantation Research Center, Research Institute for Oncology, Hematology and Cell Therapy, Tehran University of Medical Sciences, Iran

**Keywords:** Chronic myeloid leukemia, Chemoresistance, Tyrosine kinase inhibitors, long non-coding RNAs, microRNAs

## Abstract

Chronic myeloid leukemia (CML), is a myeloproliferative disease characterized by unregulated growth of blood forming cells in bone marrow and blood. The t(9;22)(q34;q11.2) translocation, which results in the formation of a hyperactive tyrosine kinase (*BCR-ABL*), is a hallmark of this disorder. Tyrosine kinase inhibitors such as imatinib has shown a great promise in reduction of CML cells. However, development of resistance to tyrosine kinase inhibitors has raised a great clinical concern about their future applications. Recently, non-coding RNAs, have shown to play significant regulatory roles in development of chemoresistance in CML cells. Discovering the underlying mechanisms of these non-coding RNAs might provide new opportunities for treating chemo-resistant forms of CML. These non-coding RNAs could be considered valuable therapeutic targets if they are found to play a role in the development of chemoresistance in CML cells. We mentioned the identified non-coding RNAs in development of chemoresistance in CML cells.

## 1 Introduction

Chronic myeloid leukemia (CML), also known as chronic myelogenous leukemia, is a type of haemopoietic stem cell disease associated with a chromosomal translocation t(9;22) (q34;q11) (Philadelphia translocation) in hematopoietic stem cells [[1], [2], [3], [4]]. Such translocation finally results in the formation of a new genetic sequence comprising of ABL gene (chromosome 9) and BCR gene (chromosome 22). The newly formed fusion gene (*BCR-ABL*), codes for a tyrosine kinase with a high activity level [3]. The molecular pathogenesis of CML is well understood. However, the mechanisms driving its progression remain poorly defined and require further investigation.

Generally, the main goal in treatment of CML is destroying CML cells that have *BCR-ABL* fusion gene [5]. Targeted therapy using tyrosine-kinase inhibitors (TKIs), is the standard approach in CML’s treatment [6]. In addition to targeted therapy, which selectively inhibits the activity of the *BCR-ABL* tyrosine kinase in CML cells, chemotherapy and other chemical agents are alternative approaches to reduce the number of CML cells by either killing them or inhibiting their growth and division [7]. TKIs have become the preferred treatment for CML due to their significant impact on disease progression and their ability to improve patient survival rates [8,9]. Despite the remarkable success of TKI inhibitors in treating CML, the development of resistance remains a major challenge in managing these patients [[10], [11], [12], [13]]. Such drug-resistance could be intrinsic or formed during time of treatment. So far, various factors have been proposed to play a role in developing TKIs resistance in CML cells [[14], [15], [16], [17]]. Some of the recognized factors and signaling pathways involving in this process are summarized in a review paper by Moraes et al. [18]. Long non-coding RNAs (lncRNA) and microRNAs (miRNAs), are two main non-coding RNAs, which are proposed to have substantial regulatory roles in establishment of drug-resistance leukemia cells [[19], [20], [21], [22], [23], [24], [25]].

Non-coding RNAs (ncRNAs), including microRNAs (miRNAs), long non-coding RNAs (lncRNAs), and circular RNAs (circRNAs), are emerging as significant contributors to chemoresistance in Chronic Myeloid Leukemia (CML). CML, driven by the BCR-ABL fusion gene, is typically treated with tyrosine kinase inhibitors (TKIs) like imatinib, but resistance remains a major challenge.

MiRNAs regulate gene expression and have been linked to chemoresistance by targeting critical pathways like apoptosis and cell survival. For example, miR-155 and miR-21 promote resistance, while miR-34a's downregulation is associated with TKI resistance. LncRNAs, such as HOTAIR and MALAT1, influence drug resistance by modulating gene expression and interacting with miRNAs. These lncRNAs also regulate cellular processes like proliferation and apoptosis, contributing to CML progression and drug resistance.

CircRNAs, which act as sponges for miRNAs, also play a role in chemoresistance, with circHIPK3 being one example. NcRNAs affect key signaling pathways (e.g., PI3K/Akt, MAPK) and impact leukemia stem cell properties, drug efflux, and metabolism, all contributing to TKI resistance.

Overall, ncRNAs are critical regulators in CML chemoresistance, offering potential as biomarkers and therapeutic targets to improve treatment efficacy and overcome resistance.

In this review after a short introduction of the lncRNAs and miRNAs, we through different investigations tried to show the roles of these non-coding RNAs, in emergence of drug-resistance in CML cells.

## 2 Non-coding RNAs

### 2.1 lncRNAs

Advances in sequencing technology have revealed that, although a significant portion of the human genome is transcribed into RNA molecules, only a small fraction of these RNAs encode proteins [26,27]. Based on a recent massive work on 41,204 genetic elements, only 0.1 % of these elements (56 genes), were responsible for coding proteins and the rest were non-coding RNA (ncRNA) transcripts [28]. The vast production of these ncRNAs suggests that they play diverse roles in various biological processes. Based on size, ncRNAs can be divided into two categories including small ones (< 200 nucleotides), such as miRNAs and piwi interacting RNAs (piRNAs), and long ncRNAs with a longer size (> 200 nucleotides), such as long ncRNAs or lncRNAs [[29], [30], [31]]. So far, miRNAs have been vastly investigated and many miRNAs with their functions have been recognized and evaluated in different normal and pathological conditions. However, there is a long way to understand the exact roles and functions of lncRNAs [[31], [32], [33]]. Recent studies have demonstrated associations between lncRNAs and human diseases, particularly cancers [[34], [35], [36]]. For instance, up/down regulation of some lncRNAs have been shown in liver cancer [37,38], colorectal cancer [39,40], breast cancer [41,42], glioblastoma [43,44], and leukemia [[45], [46], [47]]. These studies indicate that dysregulation of lncRNA expression can influence various cellular processes, including cell proliferation, apoptosis resistance, angiogenesis, drug-resistance, metastasis, and the silencing of tumor suppressors [37,38,48,49]. Interactions with chromatin, chromatin remodeling, and generally acting as transcription regulators are considered as the most probable functions of lncRNAs [[50], [51], [52], [53]]. During years after their discovery and their more detailed characterizations, different mechanisms of action have been identified for lncRNAs (Table 1) [54,55].

**Table 1:** Proposed lncRNAs mechanism of actions with actual examples.

lncRNA function	Description	Example	Reference
Signal lncRNA	Regulation of transcription with response to different stimuli	*Kcnq1ot1, MALAT1*	[56,57]
Decoy lncRNAs	Blocking binding sites or having a binding site for a specific factor (miRNA, transcriptional factor, etc.), attaching to them and finally decrease their availability.	*H19, ROR*	[58,59]
Scaffold lncRNAs	Function as a platform: provide a structure for gathering different factors and forming a multi-component complex (e.g. ribonucleoprotein complexes).	*HOXA11-AS, GClnc1*	[60,61]
Guide lncRNAs	Guiding the functional platforms and complexes like ribonucleoprotein complexes into the action sites.	*Fendrr*	[62]
Enhancer lncRNAs	Affecting DNA 3-dimensional organization and holding (tethering) functional proteins in enhancer regions of DNA.	HEIH	[63,64]
Coding short peptides	Code for short peptides that might have some functions.	–	[65,66]

The main distinguishable feature of lncRNAs that differentiate them from other cellular RNAs like miRNAs and small interfering RNAs (siRNAs), is their size. Being in huge amounts in genome [67], having no open reading frame (ORF) for coding proteins (some of the lncRNAs have been characterized with ORFs and ability to code small peptides) [68], having a poor conserved DNA sequence but a high conserved promoter sequence (only a small percentage of lncRNAs have a conserved DNA sequence among primates) [31], possessing two-six exons, polyA+ tails (in 60 % of cases), and 5 ′ cap [69,70], are some other characterized features of lncRNAs. In fact, after transcription, lncRNAs are experiencing some maturation processes, like alternative splicing and polyadenylation to become functional sequences [71]. lncRNAs have highly conserved sequences at their promoter regions and are present in nearly all body tissues and cellular compartments. This suggests that they play diverse roles in various regulatory pathways [72,73]. Currently, extensive investigations are ongoing to find out the exact lncRNAs mechanism of actions in both normal and pathological situations (especially cancers). Understanding the exact actions of these RNA molecules, their regulatory mechanisms and their final targets, enable scientists to modulate them for specific therapeutic applications [74].

### 2.2 miRNAs

microRNAs or miRNAs are another class of non-coding RNAs, which have considered as revolutionary molecules since their discovery. These groups of small RNAs with 19–24 nucleotides, have shown to play a key role in regulation of gene expression in different normal and pathological conditions [75]. miRNAs are able to bind to their messenger RNA (mRNA) targets (3′ untranslated regions of mRNAs), and consequently stop their translations into the proteins [75]. Presence in high quantities in many mammalian cells and having conserved sequences, indicate the important roles of miRNAs in various biological pathways [76,77]. Nowadays, it is recognized that most of the mammalian mRNAs are under regulation by these small RNAs [78,79]. Accordingly, miRNAs have shown to be involved in different main cellular processes such as cell division [80], cell metabolism [81], cell development [82], as well as cell carcinogenesis [83]. Their activity in cancer development and suppression, as well as their aberrant expressions in different tumor stages have been shown by various investigations [84,85]. There is a growing body of evidence, which reveals the roles of miRNAs in development of hematological malignancies, such as acute lymphocytic leukemia (ALL) [86], chronic lymphocytic leukemia (CLL) [87], acute promyelocytic leukemia (APL) [88], and CML [86,87]. In case of CML, up/down-regulation of different miRNAs in patient cells have suggested the contribution of these molecules in progression of this leukemia [89,90].

## 3 non-coding RNAs and chemo-resistant CML cells

As mentioned in the introduction, TKIs have been the first therapeutic choices for treatment of CML patients. The most famous TKIs is imatinib, which actively target the *BCR-ABL* proteins and stop the subsequent signal transduction. Imatinib, has shown a great performance in reducing the number of leukemia cells and increasing the survival rate in the patients [91]. However, in recent years, a growing number of resistances CML have been detected. These resistant cells showed different responses to treatment with imatinib, as well as other TKIs. This can be a major clinical problem for treatment of CML patients. Although, the exact mechanisms of resistance to TKIs is not clear, alterations in some cellular processes, such as epigenetic modifications, mutations in the *BCR-ABL*, copy number of the tyrosine kinase *BCR-ABL*, and abnormal expression of drug transporters, have been suggested to play a part in this phenomenon [[92], [93], [94], [95], [96], [97]].

Here, evidence of non-coding RNAs has been detected, which may provide new insights into the mechanisms underlying chemoresistance in CML. Recently, there has been a great interest in identification and study of such enigmatic factors in chemo-resistant leukemia cells. For instance, recent experiments have shown a direct role of non-coding RNAs in expression levels of ABCC2 (also known as multidrug resistant protein 2). This transporter is a member of ATP-binding cassette (ABC) transporters, which play a significant role in development of chemoresistance in different cancers [98,99]. miR-27a, miR-490–3p and let-7c are identified as expression regulators of ABCC2 [[100], [101], [102]]. Presence of these miRNAs in low or high levels can lead to a remarkable alteration in the expression of ABCC2. In addition, since imatinib is a substrate of ABC transporters, some scientists believe in the role of these transporters and their regulatory factors (non-coding RNAs), in development of chemoresistance in CML cells [94,103,104].

Another lncRNA, HOTAIR, is enhanced in CML patients and imatinib-resistant K562 cells with elevated MRP1 expression. HOTAIR facilitates drug-resistance through the stimulation of the PI3K/Akt signaling pathway [105].

Small nucleolar RNA host gene 5 (SNHG5), or U50HG is one of the well-known lncRNAs with a characterized regulatory function [106]. Abnormal expression of this lncRNA has been shown in various cancers [[106], [107], [108], [109], [110]]. In a recent experiment by Baoming et al., the expression pattern of SNHG5 and ABCC2 were evaluated in CML patients to understand the probable associations between these factors and development of imatinib-resistance [24]. Findings revealed high levels of ABCC2 transporter and SNHG5 in blood cells of CML cases and a positive correlation between the expression levels of these two elements. On the other hand, the expression levels of SNHG5 and ABCC2 were found to be high in imatinib resistant cells (K562-R). In *silico* and in *vitro* assays revealed the interactions between the SNHG5 and a miRNA called miR-205–5p Further analysis showed that such interactions, finally result in a suppression in the activity of miR-205–5p, which is a regulatory factor for controlling the expression of ABCC2 in imatinib resistant cells. After knocking-down the SGHG5 in K562-R cells, a reduced amount of imatinib resistance was observed in the cells. Accordingly, the SGHG5 as a lncRNA was introduced as a key regulatory player in developing imatinib resistant CML cells. This experiment revealed a two-step pathway. First, a decoy lncRNA suppresses a regulatory miRNA. This leads to the overexpression of an ABC transporter (ABCC2), ultimately resulting in the development of drug-resistant leukemia cells [24]. Upregulation and serving as a prognostic biomarker in acute lymphoblastic leukemia (ALL) [106], and regulating gefitinib resistance in lung adenocarcinoma cells through interactions with miR-377/CASP1 axis [111] are other recent discovered activities of SGHG5.

Maternally expressed gene 3 (MEG3), is another newly identified lncRNA that recently showed to have a role in development of imatinib resistance in CML cells. In one experiment, after checking the MEG3 expression levels in both K562 and peripheral blood cells from CML patients, a significant decrease was observed in the expression of this lncRNA in drug-resistant cells [25]. The same results were obtained after checking the MEG3 expression levels in imatinib-resistant K562 cells. Overexpression of this lncRNA in resistant cells significantly improved the apoptosis levels and reduced the cell proliferation. In addition, such overexpression showed to have a remarkable decrease in the expression of some ABC transporters (ABCG2, ABCC1 and ABCB1), and finally reversing the imatinib resistance in CML cells. Further investigations revealed the interactions between MEG3 and a regulatory miRNA (miR-21). A negative correlation between these two non-coding RNAs in the resistant cells is reported [25]. The data from this experiment further elucidated the mechanisms underlying the formation of drug-resistance CML cells, involving three key players: lncRNA MEG3, the regulatory miRNA miR-21, and ABC transporters.

Contribution of MEG3 in induction of drug-resistance in other cancers, such as lung cancer (cisplatin resistance) [99], and colorectal cancer (oxaliplatin resistance), also was shown in other studies [112]. Moreover, MEG3 was found to have other regulatory roles and miRNA targets in CML cells. For instance, Li et al., showed an abrupt expression of MEG3 and its regulatory target miR-147 in patients with accelerated and blast phases of CML [113]. In this study, over-expression of MEG3 in CML cells resulted in a significant improvement in apoptosis rate, as well as a reduction in expression of several genes like DNA methyltransferases (DNMT1, DNMT3A, DNMT3B), histone deacetylase 1 (HDAC1), methyl-CpG binding domain protein 2 (MBD2), and methyl CpG binding protein 2 (MECP2) [113]. Altogether, it seems that the MEG3 is a lncRNA with a diverse range of functionalities and more investigations are needed to disclose its unknown functions and underlying mechanisms in cancers like leukemia.

Urothelial carcinoma-associated 1 (UCA1), is another lncRNA, which found to be associated with tumor progression, metastasis and drug-resistance in different cancers [[114], [115], [116], [117]]. In a recent work, UCA1 was identified as a contributor in developing imatinib resistant CML cells [118]. This lncRNA acts as a competitive endogenous RNA (ceRNA), via binding to miR-16, and causing an increase in expression levels of ABCB1 transporter in CML cells [118]. According to the authors, modulating the activity of this lncRNA via its knocking down/out in drug-resistant CML cells can affect treatment outcomes in CML patients.

The silencing of HULC resulted in an elevation of the apoptosis-inducing caspase-3 and a reduction in the expression of c-Myc and Bcl-2. The lowering of HULC was shown to decrease PI3K/AKT phosphorylation, hence regaining IM sensitivity in K562 cells. Prior research indicated that inhibiting the PI3K/AKT pathway enhances the sensitivity of CML LSCs to treatment, hence reinforcing the assertion that HULC plays a role in imatinib resistance via elevating phosphorylation within the PI3K/AKT pathway [119].

The lncRNA NEAT1, controlled by MYC, mediates imatinib-induced apoptosis in CML cells [120].

In the study of Dai et al., it was observed that lncRNA OIP5-AS1 was substantially expressed in drug-resistant CML cells (K562/G01) and patients. The knockdown of OIP5-AS1 enhanced the sensitivity of resistant CML cells to chemotherapy by reducing chemotherapy-induced autophagy. Furthermore, OIP5-AS1 sequestered miR-30e-5p to enhance ATG12-mediated autophagy through a competing endogenous RNA (ceRNA) network, thereby augmenting the resistance of CML cells to TKI [121]. ATG12 is a crucial molecule in the autophagy process. The autophagy process primarily encompasses initiation, vesicle nucleation and elongation, fusion of autophagosomes with lysosomes, and the destruction and recovery of autophagosome contents. The autophagy process is facilitated by several autophagy-related proteins, including mTOR, ULK1, Beclin-1, LC3, ATG5, and ATG12. The ATG5-ATG12 complex associates with ATG16 to enlarge the autophagosome membrane, ultimately resulting in the formation of an autophagosome [122]. Numerous investigations have confirmed that ATG12 facilitates resistance to chemotherapy and radiotherapy [123,124]. The lncRNA MALAT1 functions as a ceRNA to enhance the production of ATG12 by sequestering miR-23b-3p, hence augmenting chemoresistance associated with autophagy in gastric cancer cells. It also indicates that alterations in ATG12 will influence autophagy levels [125].

The lncRNA MALAT1/miR-328 axis has been observed to enhance the proliferation of CML cells and confer resistance to imatinib [126]. A lot of evidence has been verified indicating that MALAT1 is associated with medication resistance [[127], [128], [129]] and cancer progression [130]. Luo et al. also discovered that the lncRNAs implicated in dasatinib resistance predominantly modulate metabolic pathways, with the main lncRNA MALAT1 associated with a favorable outcome in CML [131].

In imatinib-resistant CML cells, the established oncogene H19 and its associated miR-675 were increased, whereas the novel long non-coding RNA LNC000093 was downregulated. LNC000093 is a newly annotated lncRNA validated by other research, and further tests demonstrated that its expression is regulated by H19/miR-675 via direct binding of miR-675–5p Expression of RUNX1 in IMR cells was post-transcriptionally diminished by miR-675–5p The findings of Wong et al. indicated reduced expression of RUNX1 in IMR cells, suggesting its possible involvement in drug response. Additionally, LNC000093 may function as a ceRNA for miR-675–5p, thereby inhibiting the H19/miR-675–5p-mediated suppression of RUNX1, resulting in the de-repression of RUNX1 and increased sensitivity to TKI treatment. The diminished expression of LNC000093 in IMR cells leads to the overexpression of miR-675–5p, which inhibits cell death after imatinib treatment, resulting in the survival and resistance of CML cells to imatinib. The combined findings indicate a potential association between the LNC000093-H19/miR-675-RUNX1 pathway and imatinib resistance in CML. The extrinsic regulatory function of exosomal H19/miR-675 and LNC000093 in the bone marrow microenvironment is evidenced, indicating a significant pathway for the evolution of drug-resistance [132].

Additionally, Lin et al. assessed the expression of CBR3-AS1 in an expanded cohort, revealing that, in comparison to the control group, there was no statistically significant difference in CBR3-AS1 expression within the deep molecular response (DMR) group. Conversely, CBR3-AS1 was upregulated in the non-DMR group, thereby reinforcing their conclusion that CBR3-AS1 overexpression correlates with BCR::ABL1-independent TKI resistance in CML patients. Subsequent mRNA and bioinformatics analyses indicated that CBR3-AS1 may have a role in BCR::ABL1-independent TKI resistance in CML patients by targeting KCNA6 [133].

Apart from the mediatory action of miRNAs in development of chemoresistance in CML cells, so far different experiments revealed a direct contribution of these small regulatory RNAs in this process. As an example, miR-202 which actively targets the hexokinase 2 (HK2), was shown to be down-regulated in resistant CML cells [134]. HK2 is a key glycolysis enzyme involves in metabolism of glucose. A recent experiment showed the correlation of glycolysis in development of imatinib resistance in CML cells [135]. Accordingly, down-regulation of miR-202 in chemo-resistant CML cells indicates the indirect role of this miRNA in development of chemoresistance in these cells. Moreover, over-expression of miR-202 in resistant CML cells was shown to sensitized them to imatinib treatment. Based on these findings, miR-202 can be considered as a therapeutic target in chemo-resistant CML cells [134]. However, more investigations are needed to clarify the actual role of glycolysis, as well as the exact mechanisms behind the miR-202 mediated imatinib resistance in CML cells. miR-9 is another example of miRNAs, which showed an aberrant expression in CML and other cancers [[136], [137], [138]]. In a recent experiment, Li et al., showed a significant down-regulation of miR-9 in resistant forms of CML cells. In *vitro* experiments also revealed a reduction in chemo-sensitivity of CML cells upon inhibition of miR-9 [139]. Moreover, both in *vitro* and in *vivo* over-expression of this miRNA led to an increase in chemo-sensitivity of drug-resistant CML cells. Further analysis showed that the miR-9 has a regulatory action on the ABCB1 and there is a negative correlation between miR-9 and this ABC transporter in drug-resistant CML cells [139]. These findings indicated the regulatory role of miR-9 in treatment response of CML patients via targeting ABCB1 transporter. Other experiments also revealed the regulatory activities of miR-9 in drug-sensitivity and drug-resistance in ovarian and bladder cancers [140,141], which suggesting involvement of this miRNA in treatment responses in different cancers.

Three miRNAs (hsa-miR-28–5p, hsa-miR-129–5p, hsa-miR-543) were identified via intersection analysis. Research indicates that these miRNAs are significantly associated with the progression of leukemia. The reduction of endogenous miR-28–5p levels by dexamethasone mitigates its capacity to inhibit the increase of NDRG2′s stress response to dexamethasone [142]; miR-28–5p reinforces the concept that the inactivation of targeted genes is associated with malignant development in cancer [143]. The expressions of IL7 and hsa-miR-28–5p exhibited a negative correlation with the overall survival of CML patients [131].

Numerous miRNAs that target BCR::ABL1 transcripts have been discovered and described. Given that miR-29b is downregulated in CML patients, it was chosen for further examination, demonstrating that the overexpression of miR-29b in the K562 CML cell line results in a reduction in BCR::ABL1 expression. The 3′ UTR of BCR::ABL1 was validated as the target region for miR-29b by a luciferase experiment, and the overexpression of miR-29b prompted apoptosis and inhibited proliferation in vitro [144].

Comparable results were observed when researchers examined miR-30a and miR-424, two more lowly expressed miRNAs in the bone marrow of CML patients [145,146]. Nonetheless, they were also demonstrated to contribute to IM resistance. The overexpression of miR-424 was demonstrated to enhance the sensitivity of K562 cells to IM therapy [146].

Imatinib treatment of CML reduces miR-30a expression. The reduction of miR-30a after IM therapy likely contributes to resistance, as miR-30a represses its targets Beclin 1 and ATG5, which facilitate intrinsic apoptosis [147]. These miRNAs directly diminish BCR::ABL1 expression, and their down-regulation contributes to imatinib resistance. Consequently, restoring homeostatic levels of miR-424 and miR-30a with the administration of miRNA mimics may serve as an effective approach to alleviate BCR::Overexpression of ABL1 that exacerbates illness severity and induces therapeutic resistance [147].

Similar to the previously mentioned miRNAs, miR-320a modulates BCR::ABL1 and is downregulated in CML mesenchymal stromal cells (MSCs). Reduced levels of miR-320a were associated with reduced survival rates in CML patients. Restoring baseline levels of miR-320a can decelerate disease progression by inhibiting phosphorylation of the PI3K/AKT pathway, downstream of BCR::ABL1, hence diminishing the growth and survival of CML and enhancing the sensitivity of CML LSCs to IM [148]. miR-196b exhibited significant methylation in CML patients, elucidating its restricted expression relative to healthy controls [149]. Luciferase studies identified both BCR::ABL1 and HOXA9 oncogenes as targets of miR-196b, and further in vitro investigations validated that miR-196b could inhibit both, reducing proliferation and cell cycle progression [149].

Comparable data suggests that miR-23a is hypermethylated in CML, correlating with its reduced expression in CML patient samples and cell lines [150]. The expression of miR-23a exhibited an inverse correlation with BCR::ABL1, and the overexpression of miR-23a resulted in a reduction in BCR::ABL1, hence inducing apoptosis [150]. The data suggest that miR-196b and miR-23a function as tumor suppressors, which are epigenetically suppressed, hence promoting the evolution of CML through enhanced translation of BCR::ABL1. The introduction of hypomethylating drugs may potentially reactivate their functioning, hence diminishing disease development and resistance. The application of hypomethylating drugs in the treatment of CML has been recorded, with recent findings demonstrating a synergistic impact of the oral demethylating agent (OR-2100) in conjunction with TKIs on CML proliferation in vitro [151].

The reduction of let-7, a miRNA precursor, and the concomitant elevation of LIN28B, a transcription factor that enhances pluripotency, facilitate the self-renewal ability and stem cell characteristics of LSC, ultimately leading to relapse and resistance to therapy in CML [152].

The downregulation of miR-203 has been demonstrated to facilitate the persistence of CML LSCs by regulating their proliferation and self-renewal. It functions by targeting the 3′ UTRs of survivin and Bmi-1 transcripts. Survivin is recognized for its significant function in inhibiting apoptosis, whereas Bmi-1 is associated with the self-renewal capabilities of stem cells. By inhibiting these molecules, miR-203 obstructs the survival and renewal of LSCs, hence countering disease persistence and relapse; yet, its deregulation in CML facilitates LSC persistence [153]. MiRNA-203 enhances the sensitivity of CML cells to Imatinib and induces apoptosis, whereas miRNA-486 fosters Imatinib resistance by targeting PTEN and FOXO1 [154].

Moreover, miR-494–3p is diminished in CML LSCs and enhances the efficacy of all three generations of TKIs to induce apoptosis. It attaches to the 3′ UTR of c-MYC, restricting its translation and producing a pro-apoptotic impact that is independent of BCR::ABL1 [155].

In CML, evidence indicates that miR-21 preserves the CML LSC after therapy with IM. Simultaneous administration of antogmiR-21 and IM enhanced the effectiveness of IM and induced apoptosis in CD34+ CML cells, while leaving normal CD34+ cells unaffected [156]. The authors determined that inhibiting miR-21 enhances the effectiveness of IM on LSCs by affecting the PI3K/AKT pathway. The PI3K/AKT pathway is crucial for the fundamental functions of many stem cells, so it should not be directly inhibited [156]. Consequently, antagomir-21 in conjunction with IM therapy is an innovative approach to inhibit elevated PI3K/AKT signaling while preserving normal stem cell functionality [156]. miR-29a-3p, which is likewise increased in CML LSCs, inhibits TET2 by attaching to its 3′ UTR. TET2 plays a role in the apoptotic response to TKI therapy and functions as a tumor suppressor, especially in myeloid malignancies [157]. Consequently, antogomiR-29a-3p may mitigate therapeutic resistance by inhibiting its target, thereby restoring TET2 transcript translation and promoting apoptosis in response to TKI treatment [157].

Likewise, miR-660–5p diminishes the sensitivity of CML to IM and is upregulated in CML LSCs. miR-660–5p preserves CML LSCs by interacting with the 3′ UTR of endothelial PAS domain protein 1 (EPAS1), hence diminishing its role in modulating cellular responses to hypoxic conditions [157]. miR-378, elevated in the bone marrow of CML patients, is demonstrated to enhance the expression of many stem cell markers, including Nanog, Oct4, and c-MYC. Prior research has associated these transcription factors with myeloid lineage leukemias. Researchers elucidated the oncogenic roles of miR-378 by confirming that FUS1, a tumor suppressor, is a target of miR-378 in K562 cell lines; thus, miR-378 promotes the proliferation of CML. In summary, miR-378 enhances the self-renewal and pluripotency of LSCs in CML, hence facilitating disease persistence and resistance to treatment [158]. miR-126–3p (miR-126) facilitates disease persistence by modulating LSC dormancy and engraftment capacity. While miR-126 is downregulated in LSCs relative to healthy long-term HSCs, it is elevated within the leukemic niche, especially in endothelial cells that release miR-126 to the LSCs. Furthermore, TKI therapy results in an elevation of miR-126, hence enhancing LSC persistence and stemness. Data indicates that the inhibition or deletion of miR-126 in mice resulted in an enhanced response to TKIs and a decrease in relapse produced from LSCs [159].

The lack of miR-328 resulted in a reduction of CEBPA protein synthesis, as miR-328 typically competes with CEBPA mRNA for binding to hnRNP E2, hence facilitating the translation of CEBPA transcripts. Anticipatedly, ectopic expression of miR-328 reestablished CEBPA translation. This investigation of miR-328 uncovered an unconventional mechanism, but the scientists also identified a traditional role of miR-328 as a negative post-transcriptional regulator of PIM1, a critical molecule for the survival of CML. miR-328 exemplifies the complex nature of miRNA functionality, as it is associated not only with conventional regulatory mechanisms but has also been characterized as a decoy for a hnRNP. The restoration of baseline miR-328 levels presents therapeutic potential by not only overcoming the differentiation impediment but also potentially inducing apoptosis in CML [160].

Data indicates that AKR1C3 is significantly up-regulated in the resistant CML BMM, and its ectopic expression reduces the efficacy of IM therapy in vitro. Elevations in AKR1C3 were adequate to activate enhanced MAPK/ERK signaling. In contrast, miR-379–5p is downregulated in the BMM of CML but can bind to AKR1C3 mRNA to inhibit its translation. Nonetheless, the restoration of miR-379–5p was adequate to reinstate the efficiency of IM via antagonizing the elevated levels of AKR1C3 [161].

Likewise, miR-221 is diminished in the mononuclear cells of peripheral blood from IM-resistant CML patients compared to those that are treatment-sensitive [162]. The study revealed that ectopic expression of miR-221 reduced the expression of STAT5. Prior study established STAT5 as a target of miR-221 [163], prompting the researchers to verify the impact of elevated STAT5 levels resulting from diminished miR-221 expression. They concluded that STAT5 suppressed apoptosis while promoting proliferation. Prior research has indicated that STAT5 may facilitate the generation of reactive oxygen species (ROS) in CML, hence contributing to therapeutic resistance through the enhancement of genomic instability [164]. miR-153–3p is downregulated in imatinib-resistant CML patients, and its restoration re-sensitizes these patients to imatinib while diminishing imatinib-induced protective autophagy. The dual-luciferase experiment validated Bcl-2 as the direct target of miR-153–3p The reduction of miR-153–3p in resistant patients, together with the accompanying elevation of Bcl-2, may mitigate therapy resistance, as research on CML have demonstrated that heightened Bcl-2 might diminish apoptosis and facilitate drug-resistance [165]. A reduction in miR-153–3p facilitates protective autophagy in CML, similarly to the downregulation of miR-199a/b-5p observed in IM-resistant K562 strains. Instead of targeting Bcl-2, miR-199a/b-5p targets WNT2. Ectopic expression of miR-199a/b-5p decreased WNT2 expression, inhibited protective autophagy, and reactivated the apoptotic response of CML to imatinib therapy [166].

Conversely, miR-577, which is downregulated in mononucleated cells in the peripheral blood of CML patients, targets NUP160, a gene that encodes a component of the nuclear pore complex. Nuclear core complexes may contribute to cancer survival by facilitating medication resistance and dormancy. Dysregulated miR-577 results in elevated NUP160 levels, which have been demonstrated to desensitize CML to IM. Conversely, ectopic production of miR-577 can restore IM efficacy and reestablish appropriate control of NUP160 [167].

Researchers identified that miR-342–5p is downregulated in CML patients, although CCND1 expression levels are notably elevated. A luciferase test validated that the 3′ UTR of CCND1 was a target of miR-342–5p Altered CCND1 expression has been identified as a factor in the progression of CML by facilitating cell cycle. The authors assert that miR-342–5p functions as a tumor promoter by indirectly inhibiting BCR::ABL1 transcripts. Administration of a miRNA mimic of miR-342–5p may potentially mitigate the elevation of BCR::ABL1 and CCND1, addressing therapeutic resistance [168].

Another mechanism contributing to treatment resistance in CML is regulated by miRNAs: drug transporters. Efflux transporters enable cells to avoid treatment by emptying the chemical from the cytoplasm. The mononuclear cells in the peripheral blood of imatinib-resistant CML patients exhibited a reduction in miR-214 compared to responsive patients. Notably, miR-214 interacts with the 3′ UTR of ABCB1, which encodes a drug efflux transporter that is up-regulated in multi-drug-resistant cancers, but with minimal sequence homology. Ectopic expression of miR-214 regained therapeutic responsiveness in imatinib-resistant cell lines, presenting miR-214 as a promising strategy to combat drug-resistance in CML [169].

Other regulatory miRNAs which are recognized as participants in development of chemoresistance in CML cells are listed in Table 2.

**Table 2:** The discovered lncRNAs and miRNAs involving in chemo-sensitivity and onset of drug-resistance CML cells.

Non-coding RNA	Targeted molecule/s	Mechanism of action	Outcome	Reference
lncRNAs				
HOTAIR	PI3K/Akt	Activation	Over-expression of ABCC1 and development of chemoresistance CML cells.	[105]
SNHG5	miR–205–5p	Suppression	Over-expression of ABCC2 and development of chemoresistance CML cells.	[24,106]
MEG3	miR-21	Suppression	Over-expression of ABC transporters, reducing apoptosis and increasing the drug-resistant cell proliferation.	[25,113]
UCA1	miR-16	Suppression	Over-expression of ABCB1 and development of chemoresistance CML cells.	[118]
ULC	PI3K/Akt, c-Myc, Bcl-2	Activation	Decreasing imatinib sensitivity in CML cells via activation of PI3K/Akt signaling pathway.	[119]
NEAT1	–	–	Down-regulation of NEAT1 results in over-expression of ABCG2 and development of chemoresistance CML cells.	[22,120]
OIP5-AS1	miR-30e-5p/ATG12 Axis	Activation	OIP5-AS1 sequestered miR-30e-5p to enhance ATG12-mediated autophagy through a ceRNA network, thereby augmenting the resistance of CML cells to TKI.	[121]
MALAT1	miR-328	Activation	Over-expression of miR-328 and enhance the proliferation of CML cells and confer resistance to imatinib.	[126]
H19	miR-675	Activation	Over-expression of miR-675 and enhance the proliferation of CML cells and confer resistance to imatinib.	[132]
LNC000093	miR–675–5p	Suppression	Expression of RUNX1 in IMR cells was post-transcriptionally diminished by miR–675–5p	[132]
*CBR3-AS1*	KCNA6	Activation	Over-expression of *CBR3-AS1* and enhance the proliferation of CML cells and confer resistance to imatinib.	[133]
miRNAs				
miR-202	HK2	Suppression	Down regulation of miR-202 causes up-regulation of HK2 which consequently leads to chemoresistance CML cells.	[134]
miR-9				[[136], [137], [138]]
miR-3142	PTEN	Suppression	Up-regulation of miR-3142 results in down-regulation of PTEN, activation of PI3 kinase pathway and finally causes drug-resistance CML cells.	[139]
miR–28–5p	NDRG2	Suppression	The reduction of endogenous miR–28–5p levels by dexamethasone mitigates its capacity to inhibit the increase of NDRG2′s stress response to dexamethasone.	[142]
miR-29b	BCR::ABL1	Suppression	Low expression levels of miR-29b cause up-regulation of BCR::ABL1 and progression toward drug-resistance CML cells.	[144]
miR-424	BCR::ABL1	Suppression	The overexpression of miR-424 was demonstrated to enhance the sensitivity of K562 cells to IM therapy.	[146]
miR-30a	Beclin 1/ATG5	Suppression	miR-30a represses its targets Beclin 1 and ATG5, which facilitate intrinsic apoptosis.	[147]
miR-320a	PI3K/AKT	Suppression	Low expression levels of miR-320a cause up-regulation of PI3K/AKT and development of resistance to therapeutic agents in CML cells.	[148]
miR-196b	BCR::ABL1/HOXA9	Suppression	miR-196b could inhibit BCR::ABL1/HOXA9, reducing proliferation and cell cycle progression.	[149]
miR-23a	BCR::ABL1	Suppression	Low expression levels of miR-23a cause up-regulation of BCR::ABL1 and development of treatment-resistance CML cells.	[150]
let-7	LIN28B	Activation	Low expression levels of let-7 cause up-regulation of LIN28B and development of chemoresistance CML cells.	[152]
miR-203	Survivin/ Bmi-1	Suppression	Low expression levels of miR-203 cause up-regulation of Survivin/ Bmi-1 and development of resistance to chemotherapy in CML cells.	[153]
miRNA-486	PTEN/ FOXO1	–	Over expression levels of miR-486 cause development of resistance to pharmacological agents in CML cells.	[154]
miR–494–3p	c-MYC	Activation	Over expression levels of miR–494–3p cause up-regulation of c-MYC and development of resistance to anticancer drugs in CML cells.	[155]
miR-21	PI3K/AKT	Suppression	Over expression levels of miR-21 cause down-regulation of PI3K/AKT and development of resistance to TKIs in CML cells.	[156]
miR-29a-3p	TET2	Suppression	Over expression levels of miR-29a-3p cause down-regulation of TET2 and development of resistance to targeted therapies in CML cells.	[157]
miR–660–5p	EPAS1	Suppression	Over expression levels of miR–660–5p cause down-regulation of EPAS1 and development of drug-resistant CML cells.	[157]
miR-378	FUS1	Suppression	Over expression levels of miR-378 cause down-regulation of FUS1 and emergence of drug-resistance CML cells.	[158]
miR–126–3p	Modulates LSC dormancy and engraftment capacity	Activation	Over expression levels of miR–126–3p cause acquisition of drug-resistance CML cells.	[159]
miR-328	PIM1	Suppression	Low expression levels of miR-328 cause down-regulation of P1M1 and formation of drug-resistance CML cells.	[160]
miR–379–5p	AKR1C3	Suppression	Low expression levels of miR–379–5p cause up-regulation of AKR1C3 and evolution of drug-resistance CML cells.	[161]
miR-221	STAT5	Suppression	Low expression levels of miR-221 cause up-regulation of STAT5 and onset of drug-resistance CML cells.	[163]
miR–153–3p	Bcl-2	Suppression	Low expression levels of miR–153–3p cause up-regulation of Bcl-2 and establishment of drug-resistance CML cells.	[165]
miR-199a/b-5p	WNT2	Suppression	Low expression levels of miR-199a/b-5p cause up-regulation of WNT2 and progression toward drug-resistance CML cells.	[166]
miR-577	NUP160	Suppression	Low expression levels of miR-577 cause up-regulation of NUP160 and development of resistance to therapeutic agents in CML cells.	[167]
miR–342–5p	CCND1	Suppression	Low expression levels of miR–342–5p cause up-regulation of CCND1 and development of treatment-resistance CML cells.	[168]
miR-214	ABCB1	Suppression	Low expression levels of miR-214 cause up-regulation of ABCB1 and development of chemoresistance CML cells.	[169]
miR-15a	BCR::ABL1	Activation	Expression regulated by BCR–ABL is associated with CML progression and imatinib resistance.	[170]
miR-130b	BCR::ABL1	Suppression	Expression regulated by BCR–ABL is associated with CML progression and imatinib resistance.	[170]
miR-145	BCR::ABL1	Activation	Expression regulated by BCR–ABL is associated with CML progression and imatinib resistance.	[170]
miR-16	BCR::ABL1	Suppression	Expression regulated by BCR–ABL is associated with CML progression and imatinib resistance.	[170]
miR-26a	BCR::ABL1	Suppression	Expression regulated by BCR–ABL is associated with CML progression and imatinib resistance.	[170]
miR-146a	BCR::ABL1	Suppression	Expression regulated by BCR–ABL is associated with CML progression and imatinib resistance.	[170]
miR-29c	BCR::ABL1	Suppression	Expression regulated by BCR–ABL is associated with CML progression and imatinib resistance.	[170]
miR-224	ST3GAL IV	Suppression	miR-224 and let-7i modulate the proliferation and chemo-sensitivity of CML cells, likely by targeting ST3GAL IV.	[171]
miR–493–5p	IL-8	Suppression	Over-expression of miR–493–5p and consequent inhibition of IL-8 can help to overcome imatinib resistance in CML cells.	[172]
miR-181a	Abi-1	Suppression	Up-regulation of miR-181a is a reason for suppression of Abi-1, increased amounts of integrin α4, abruption in Fak/Akt/Erk signaling pathway, and finally imatinib-resistance in CML cells.	[173]
miR-181c	ST8SIA4	Suppression	Low expression levels of miR-181c results in deregulation of ST8SIA4 expression and MDR progression in CML cells.	[174]
miR-212	ABCG2	Suppression	Low expression levels of miR-212 cause up-regulation of ABCG2 and development of resistance to chemotherapy in CML cells.	[175]

Abbreviations: HOTAIR: HOX transcript antisense RNA, SNHG5: small nucleolar RNA host gene 5, MEG3: maternally expressed gene 3, UCA1: urothelial carcinoma-associated 1, NEAT1: nuclear paraspeckle assembly transcript 1, ATG12: autophagy related 12, MALAT1: metastasis associated lung adenocarcinoma transcript 1, HK2: hexokinase 2, IL-8: interleukin 8, Abi-1: Abelson interactor protein 1, PTEN: phosphatase and tensin homolog. MDR: multiple drug-resistance, ST8SIA4: ST8 alpha-N-acetyl-neuraminide alpha-2,8-sialyltransferase 2, EPAS1: endothelial PAS domain protein 1.

## 4 Conclusion

**Fig. 1:**
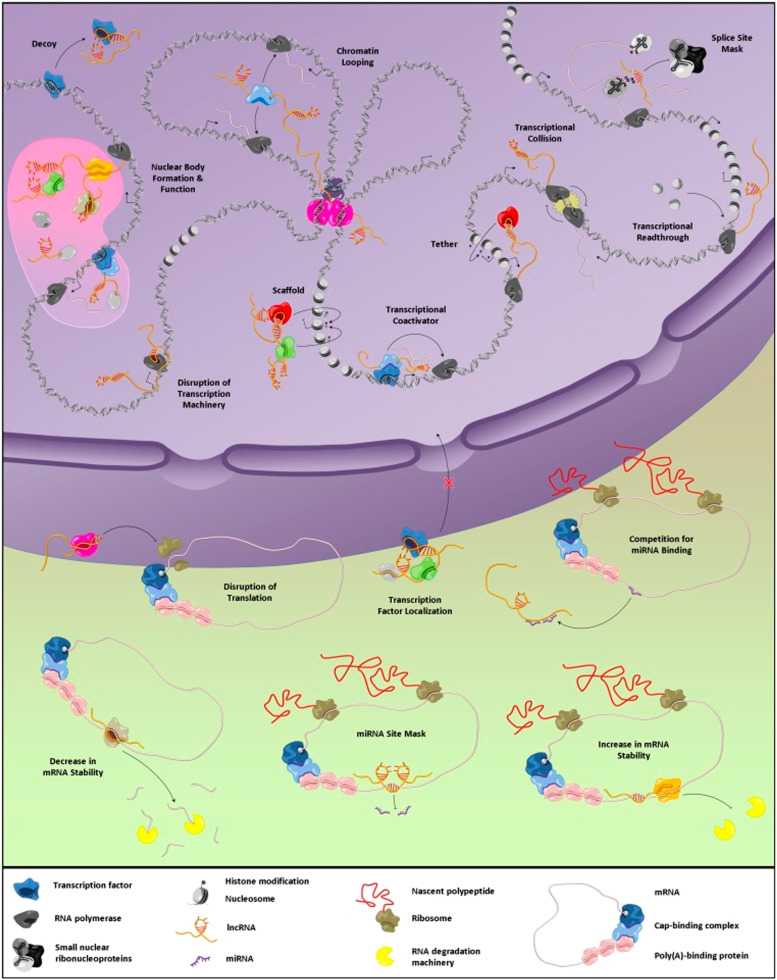
Different identified functions of lncRNAs. Reused with permission [29].

Numerous experiments have demonstrated the role of lncRNAs in the progression of various human cancers. However, their exact mechanisms of action remain incompletely understood. Research into these enigmatic molecules is still in its early stages. In case of CML, different experiments have tried to evaluate the expression levels of non-coding RNAs in patient cells and suggested the impacts of their differentiated expression levels on different aspects like cancer progression, apoptosis and their responses to anti-tumor drugs [176,177]. Nowadays, researchers have focused on finding the exact underlying mechanisms of non-coding RNAs in development of multiple drug resistance in CML cells. One part of these extensive researches was performed on the role of these non-coding RNAs in development of imatinib resistant CML cells. Recently, with increasing the number of imatinib resistance in CML patients, especially for those at the advanced stage, a great concern has been formed about the limitation in application of imatinib and final clinical outcomes in the patients. Here we mentioned several experiments showing the impacts of non-coding RNAs including lncRNAs and miRNAs in development of chemo-resistant CML cells. Based on the findings, it can be concluded that the impacts of lncRNAs is mainly through disruption of miRNAs regulatory pathways and increasing the expression of ABC transporters, which finally lead to pumping tumor suppression drugs out of the CML cells. Apart from the mediatory roles of miRNAs in this phenomenon, abrupt expression of these small molecules can itself be a trigger for development of chemoresistance in CML cells. In the near future, once the exact mechanisms of these regulatory molecules are understood through high-throughput assays, they could serve as prognostic markers or even therapeutic targets. In case of lncRNAs, it seems that these molecules have a great potential for development of novel therapeutics. For instance, with reducing the expression levels of one lncRNA, which found to be involved in drug resistance development in CML cases, the treatment could be more effective in the patients. On the other hand, advances in delivery techniques could open new avenues for the development of lncRNA-interfering agents as innovative therapeutics against drug-resistant hematopoietic malignancies (Fig. 1).

## CRediT authorship contribution statement

**Laya Ghadyani nejhad:** Writing – original draft. **Mahsa Sohani:** Writing – review & editing. **Nasrin Alizad Ghandforoush:** Methodology. **Mohsen Nikbakht:** Supervision. **Saeed Mohammadi:** Supervision. **Mohammad Vaezi:** Supervision. **Shahrbano Rostami:** Supervision, Conceptualization. **Bahram Chahardouli:** Supervision, Conceptualization.

## Declaration of competing interest

The authors report there are no competing interests to declare.

## Appendix Supplementary materials

Image, application 1

Image, application 2

Image, application 3
