# Supplementary figures and images for: Non-coding RNAs: Emerging contributors to chemoresistance in chronic myeloid leukemia
Source: Leuk Res Rep. 2025 May 9;23:100513. doi: 10.1016/j.lrr.2025.100513 (PMC12144514; doi:10.1016/j.lrr.2025.100513)

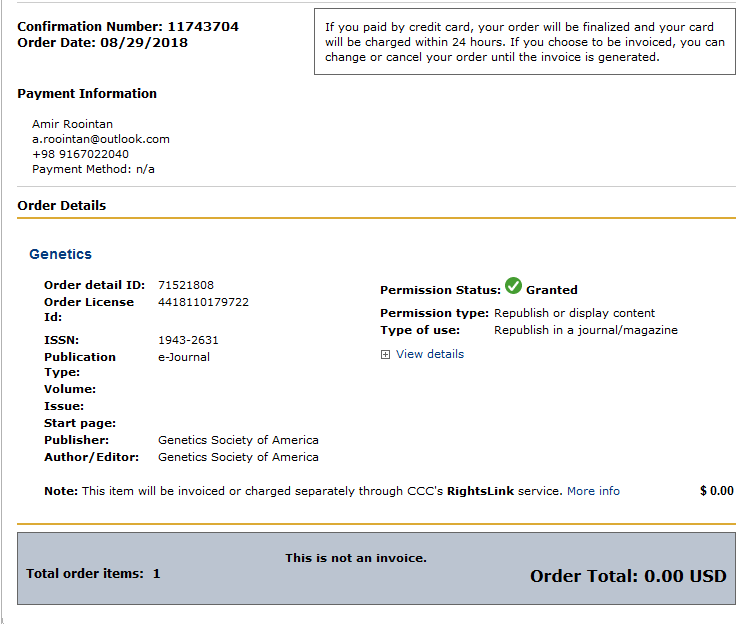

Supplement: Supplementary file 1 [file mmc1.zip › mmc1.PNG]

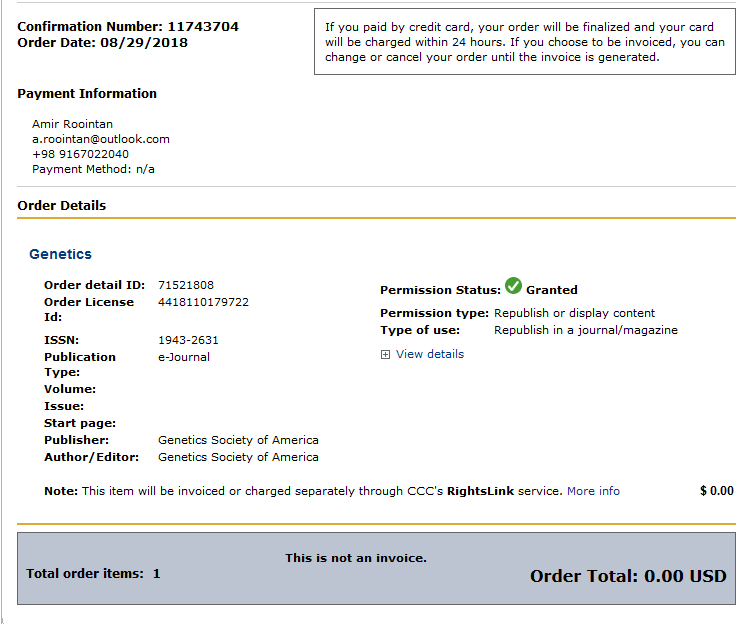

Supplement: Supplementary file 2 [file mmc2.docx]

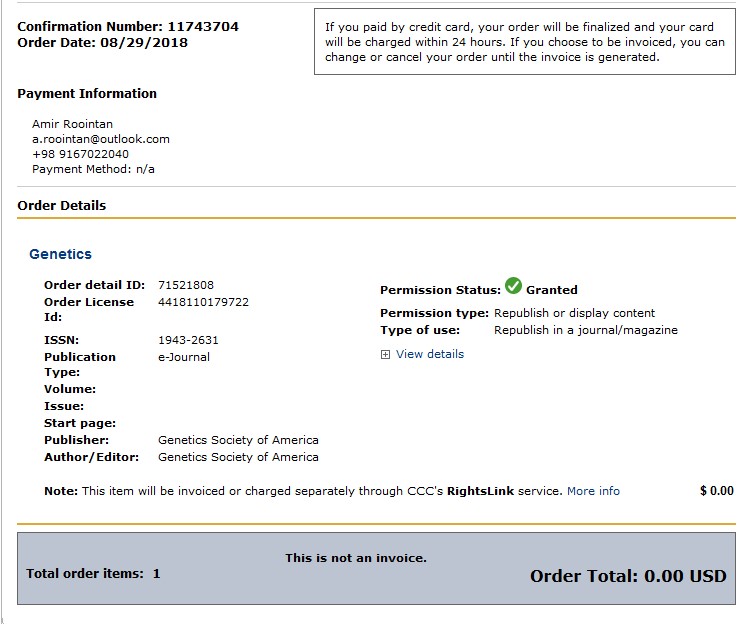

Supplement: Supplementary file 3 [file mmc3.zip › mmc3.jpg]
